# More Than Just Kibbles: Keeper Familiarity and Food Can Affect Bonobo Behavior

**DOI:** 10.3390/ani13030410

**Published:** 2023-01-26

**Authors:** Marta Caselli, Emilio Russo, Jean-Pascal Guéry, Elisa Demuru, Ivan Norscia

**Affiliations:** 1 Department of Life Science and System Biology, University of Torino, Via Accademia Albertina 13, 10123 Torino, Italy; 2 La Vallée des Singes, 86700 Romagne, France; 3 Laboratoire Dynamique du Langage, CNRS-UMR 5596, Université de Lyon, 14 Avenue Berthelot, 69363 Lyon, France; 4 ENES Bioacoustics Research Lab, CRNL, CNRS-UMR 5292, InsermUMR_S1028, Université de Saint-Etienne, 21, rue du Dr. Paul Michelon, 42100 Saint-Etienne, France

**Keywords:** animal welfare, food, keeper familiarity, social behavior, *Pan paniscus*

## Abstract

**Simple Summary:**

Food availability can affect animal welfare, but it is not the only factor in play. Caregivers also affect welfare, and this aspect may especially apply to bonobos, the great apes that (with chimpanzees) are closest to us. In a bonobo group (17 individuals; La Vallée des Singes, France), we video-recorded behaviors possibly expressing positive emotions (e.g., play) and negative emotions (e.g., aggression or displacement anxiety activities), requests (gestures) and affiliation (e.g., sociosexual interactions). Within a few minutes around food provisioning, we determined behavioral frequencies with more/less familiar keepers and with different food types (kibbles/fruits/vegetables) or no food. More familiar keepers—regardless of food—were linked to more sociosexual contacts, which are probably used to contain excitement (more familiar keepers are strongly associated with forthcoming food). More familiar keepers—when distributing fruits/vegetables—were linked to more gestures, probably used to request and catch fruit items first. Preferred food increased aggression, with no keeper effect, probably because a highly valued resource (kibbles, not catchable by hand) was at stake. Play did not vary possibly because it works over longer time windows. Bonobo welfare may be improved by considering more than just food and that great apes are more similar to humans than to other animals.

**Abstract:**

The welfare of captive animals relies on numerous factors. Keepers can affect animals’ welfare and this could especially apply to emotionally and cognitively complex species, such as great apes. We collected video data over three months on 17 bonobos (La Vallée des Singes, France) and extracted five behaviours (play, aggression, anxiety, gestures, sociosexual interactions) —during two-minute slots—under three conditions: keeper-present/food-unavailable; keeper-present/food-available; keeper-absent/food-unavailable. We ran generalized linear models to investigate whether behavioral frequencies were affected by food presence/quality and keeper familiarity. Anxiety-related behaviors increased when the keeper was present and in absence of food, due to food expectation. Sociosexual interactions increased in presence of more familiar keepers and in absence of food, maybe to decrease the tension around food. Gestures increased in presence of more familiar keepers and with low-quality food, which was provided in large ‘catchable‘ pieces. Aggression levels increased with high-quality food with no effect of keeper. Play behavior was not affected by any variable. Hence, bonobos were affected not just by food but also by keeper features. Considering multiple variables in the ‘welfare equation’ can improve captive management and increase the well-being of bonobos, a species that is much closer to humans than to other non-human animals.

## 1. Introduction

The notion of welfare plays a key role in determining the quality of life of captive animals [1]. Both internal (e.g., anxiety levels) and external (e.g., type of captive management) factors concur in influencing animal well-being [2,3]. As concerns the influence of external factors, most studies of the last half-century have focused on the environment where animals are housed [4]. Only in the past 20 years have researchers started to consider other external factors, such as the keeper (including how they relate and how familiar they are to animals) and food, as possible elements that can also influence animal welfare [4,5,6]. 

For example, it has been well documented that in captivity the interactions between keepers and mammals can impact their fearfulness (e.g., maned wolf, *Chrysocyon brachyurus* and cheetah, *Acinonyx jubatus jubatus* [7]; crested macaque, *Macaca nigra* [8]). In this view, the way keepers relate to animals can be considered as a sort of enrichment that may increase animal welfare [9]. Moreover, animals may discriminate between different keepers [6] and their behavior can vary depending on the number of keepers who manage them, and the time keepers spend with them [4,6]. Finally, animal well-being can be affected by the quantity and quality of food and by the way it is provisioned [5]. For example, when food access is restricted to reduce the risk of obesity, animals can show negative behaviors [5]. 

In primates—spanning lemurs, monkeys and apes—keepers and food can influence different types of social behavior [4,10]. When the keeper spends more time with primates (e.g., by simply being present), their level of familiarity increases and there may be an increase of affiliative behavior and/or a decrease of agonistic displays and anxiety behavior (e.g., common marmosets, *Callithrix jacchus* [11]; chimpanzees, *Pan troglodytes* [12]; lowland gorillas, *Gorilla gorilla* [13]; hamadryas baboon, *Papio hamadryas* [14]). 

Moreover, food limitation in primates can lead to an increase of food seeking and related arousal, which can lead to the expression of food anticipation anxiety (e.g., via scratching [15,16,17] and yawning:[17]) and/or to an increase of conflicts over food resources (e.g., hamadryas baboons [14]; chimpanzees [18]). Additionally, the type of food (low/high caloric intake) can impact primate behavior. For example, depending on the species, high-quality food can lead to an increase of affiliation, including play (e.g., Hanuman langur, *Semnopithecus entellus* [19]; squirrel monkeys, *Saimiri oerstedi* [20]), or to an increase of intra-group competition leading to anxiety increase (chimpanzees [21,22]). 

In great apes, requesting gestures can be influenced by keeper and food, as gestures can be used to get attention from another individual (either conspecific or not) and to obtain particularly wanted resources, including food [23,24,25]. 

The bonobo (*Pan paniscus*)—together with the chimpanzee—is the species that is phylogenetically closest to humans (with the *Homo-Pan* divergence dating around seven million years ago [26]). Bonobos are generally considered as a tolerant species, with low aggression levels and usually interacting peacefully with unfamiliar subjects [27,28,29,30]. However, aggression can increase around food, especially if its quality is high [31].

Bonobos use play and sociosexual contacts as an important part of affinitive behaviors [32,33]. Play behavior in captivity can increase before the beginning of food distribution while individuals await food [33]. Sociosexual interactions can be expressed in tense situations and can work in reducing anxiety [34,35,36]. Finally, bonobos possess a variety of requesting gestures [37] and they can show a higher number of requesting gestures to familiar than unfamiliar people [38]. 

Based on this framework, we formulated the following predictions on the effect of keeper and food on different behavioral categories.

*Anxiety behavior* (Prediction 1): because in primates the interaction with keepers can have a positive effect on individuals [9] and bonobos are a xenophilic species [39], we expect to find a reduction of anxiety behaviors in presence of the keeper (Prediction 1a) but no effect of keeper familiarity (Prediction 1b). In bonobos, arousal peaks before the beginning of food distribution, therefore we expect anxiety behaviors related to food anticipation arousal to be more frequent in absence than in presence of food (Prediction 1c). 

*Sociosexual interactions* (Prediction 2): because in primates the interaction with keepers can have a positive effect on individuals [9] and bonobos can use sociosexual behavior to reduce arousal (e.g., post-conflict [40]), we expect to find a reduction of sociosexual behaviors in presence of the keeper (Prediction 2a), but no effect of keeper familiarity (Prediction 2b). As sociosexual behaviors in bonobos are usually highest after food distribution and high-quality food can induce competition-related arousal [31,41], we expect sociosexual behaviors to increase in presence of food, especially if the food is of high quality (Prediction 2c). 

*Requesting gestures* (Prediction 3): because imperative gestures (*stricto sensu*) are by definition directed to another individual [24,25] and bonobos can increase their requesting gestures when the recipient is familiar to them [38], we expect gestures to increase in presence of the keeper (Prediction 3a), especially if the keeper is highly familiar (Prediction 3b). Given that imperative gestures are usually used to obtain wanted resources [24,25], we expect them to be used more frequently in the presence of food, especially when the food is of high quality (Prediction 3c). 

*Play behavior* (Prediction 4): given that keeper presence and familiarity can increase affiliation in great apes (e.g., chimpanzees [12]) and that one of the functions of play in bonobos is to increase social affiliation [42], we expect play to increase in presence of the keeper (Prediction 4a), especially if the keeper is more familiar (Prediction 4b). Previous studies reported that in cases of scheduled feeding sessions, captive bonobos play more in the pre-feeding period (before food is made available) than when food is distributed (when it is made available), possibly because play may help buffer mild anxiety [33]. Hence, we expect play behavior to be higher in absence than in presence of available food (Prediction 4c).

*Agonistic behavior* (Prediction 5): despite the positive effect that keepers may have on animals [9], given the low aggression levels of bonobos [30]we expect to find no appreciable effect of keeper presence (Prediction 5a) and familiarity (Prediction 5b) on the frequency of agonistic events. However, given that in primates (including bonobos) aggression can increase when highly wanted food resources are at stake [31], we expect aggressive events to be more frequent in presence of food, particularly when the food is of high quality (Prediction 5c). 

## 2. Materials and Methods

### 2.1. Ethical Statements

This study is purely observational, so no approval was required from the authors’ institution. In addition, the authors read the journal policy regarding the animal ethics and confirm the compliance of the study.

### 2.2. Study Site and Group

The study was conducted on a stable bonobo group housed at La Vallée des Singes (Romagne, France). The bonobo area was composed of an indoor enclosure (~500 m^2^) and a wooded external island (~1 ha). With the exception of bad weather days, when animals were kept in the indoor enclosure, bonobos were free to move from indoor to outdoor (from around 9:30 a.m. to around 6:30 p.m.). The group was composed of 17 individuals (age range: 2–51 years; mean ± SE: 15.706 ± 3.045) and included all age classes (adults: three males and six females, age: ≥12 years; juveniles: two males and two females, age: 6–10 years; infants: one male and two females, age: 3–5 years; and one weaning female, 2 years old; see Table S1). Group management was entrusted to one out of four keepers (two men and two women) per day. Two keepers (a man and a woman) had been working with bonobos regularly (at least 5 days/week) for at least five years whereas two others (a man and a woman) had been working with bonobos occasionally (1 or 2 days/week) for less than five years. Animals were fed by keepers four times per day (11:30 a.m., 2:30 p.m., 3:45 p.m. and 5:00 p.m.). 

### 2.3. Data Collection and Operational Definitions

**Figure 1:**
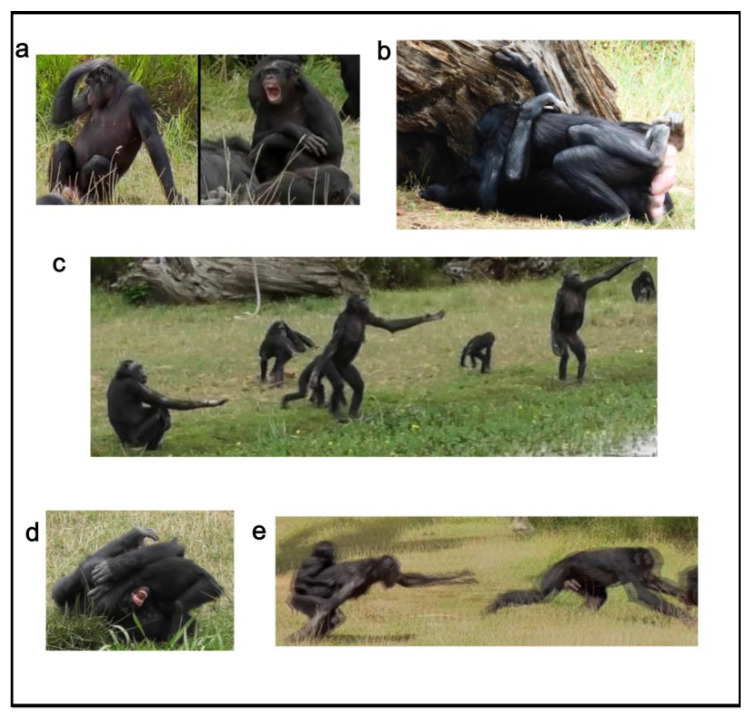
Behavioral patterns considered for this study. (**a**) Anxiety behavior (scratching and yawning); (**b**) sociosexual behavior (genito-genital rubbing between two females); (**c**) requesting gestures; (**d**) play behavior; (**e**) agonistic behavior.

Data were collected daily (July–September 2019, from around 10:00 a.m. to 6:00 p.m.) via video-recordings, when the bonobos were outdoor. Behavioral data were extracted from videos—via the all occurrences sampling method [43]—on arousal/anxiety related behavior (i.e., scratching and yawning), sociosexual behavior, play behavior, agonistic behavior and requesting gestures (see Table S2 for a full description of these behavioral categories; Figure 1). Video-coding was performed by one person (E.R.). The coder underwent a period of training with the trainer (E.D.) before starting the video-analysis. The training period ended when the observer and the trainer reached 100% agreement. 

We randomly selected two-minute time slots and we extracted data under three different conditions: (1) bonobos could see the keeper with food (in a basket) either just before or during the food distribution session (when bonobos had finished the first ration of food and were waiting for the next one), but food was not yet available in their enclosure (keeper-present/food-unavailable); (2) bonobos could see the keeper and food was available (keeper-present/food-available); (3) the keeper was absent and no food was available (keeper-absent/food-unavailable). The condition of keeper-absent/food-available did not occur in the visible part of the enclosure and/or in the foreseen time window. In total, we were able to analyze 118 video sequences, equally distributed across conditions and food and keeper features, for a total of 236 min. 

To check for the possible effect of keeper familiarity on bonobo behavior, we labelled the keepers (a man and a woman) regularly working with bonobos for at least 5 years as ‘more familiar’. We labeled the others (a man and a woman, occasionally working with bonobos for less than 5 years) as ‘less familiar’. We also checked for the possible role of food type in shaping bonobos’ behavior. To do this we considered the following categories: absence of food; large, low-quality food (i.e., fruit and vegetables cut in large pieces, ~23 kcal. × 100 g), and small, high-quality food (St Laurent© small kibbles for Old World primates ~370 kcal. × 100 g). For information on fruit and vegetable caloric intake we used the FoodData Central by the U.S. Department of Agriculture. Full details on food caloric intake are reported in Table S3. All keepers distributed food by launching food pieces to animals. 

### 2.4. Statistical Elaboration

For each two-minute time window/condition, we recorded the occurrence of all target behavioral patterns (anxiety behavior, sociosexual behaviors, requesting gestures, play behavior, agonistic behavior) performed by all bonobos. 

We used the R-function *fitdist* [44] to plot and compare if the count data followed a Poisson or a negative binomial distribution [45,46]. In addition, we compared the two Bayesian Information Criteria (BIC corrections) (Poisson and negative binomial), and we checked for the lowest BIC [47] and selected the negative binomial distribution. 

We ran a first Generalized Linear Model (GLM ) (GLM_a_; N = 84) on each target behavior (numeric variables; anxiety behavior: GLM_1_; sociosexual behavior: GLM_2_; requesting gestures: GLM_3_; play behavior: GLM_4_; agonistic behavior: GLM_5_) to investigate the impact of the keeper presence and the type of food on bonobos’ social behavior and dynamics. For all five GLMs, the following fixed factors were included: keeper presence/absence (factor; 0 = absence; 1 = presence) and type of food (factor; 0 = absence of food; 1 = food with low caloric intake; 2 = food with high caloric intake).

If the variable presence/absence of the keeper had a significant effect on the target variable, we proceeded with a second GLM (GLM_b_, N = 56) to test whether the familiarity of the keeper could impact social behavior. Here, the keeper sex was included as control variable. Hence, we included the keeper sex (factor; 1 = man; 2 = woman) and familiarity (factor: 1 = more familiar; 2 = less familiar) as fixed factors. If the variable familiarity had a significant effect, we ran a third GLM (GLM_c_, N = 56) with the keeper identity as fixed factor. This factor was tested in a separate model because sex and familiarity were strongly dependent from identity (Cramer’s V between sex and identity = 1; Cramer’s V between familiarity and identity = 1). 

All the GLMs were fitted in R [48] by using the function *glm.nb* of the R-package *MASS* [49]. At first, we performed the likelihood ratio test [50] (analysis of variance with argument *Chisq*) to verify if the full model significantly differed from the null model [51]. Subsequently, via the R-Function *drop1*, we extracted the *p* values of predictors basing on the likelihood ratio test between the full and the null model [52]. When the multinomial predictor ‘type of food’ had a significant effect, we performed all pairwise comparisons with the Tukey test [53] using a multiple contrast package (*multcomp*). In this case, we reported *p* values, estimate (Est), standard error (SE), and the Z value, adjusted for the Bonferroni correction. We reported the best effect size of each variable, calculated via the function *effectsize* from the package *effectsize*.

## 3. Results

**Figure 2:**
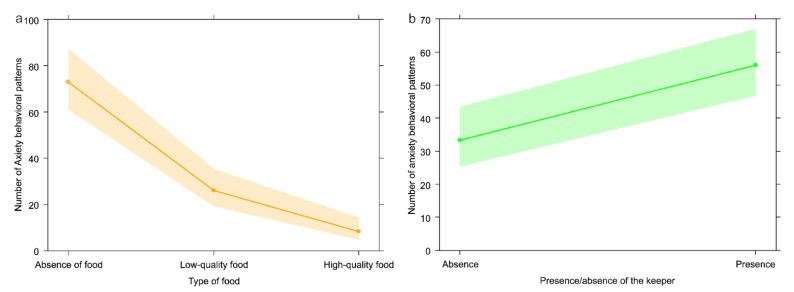
Effect plot of the variables with a significant effect on anxiety behavior. The number of anxiety behavioral patterns (y-axis) was highest (**a**) in absence of food (x-axis) and (**b**) in presence of the keeper.

The full model (GLM_1a_—target variable: anxiety-related behavior) including all fixed factors (food type and presence/absence of the keeper) was found to significantly differ from the null model (likelihood ratio test: χ^2^ = 45.835; *df* = 3; *p* < 0.001). The keeper presence had a significant effect on the target variable and was associated with an increase of anxiety behavior (*p* = 0.002; Table 1; Figure 2b). Moreover, food presence regardless of the type had a significant effect on expression of the target behavior (*p* < 0.001; Table 1; Figure 2a). Anxiety behavior was higher in absence of food and in presence of fruit and vegetable (low-quality food) than in presence of kibbles (high-quality food) (Tukey test: low-quality food vs. absence of food, Est = −1.031, *SE* = 0.187, Z = −5.525, *p* < 0.001; high-quality food vs. absence of food, Est = −2.161, *SE* = 0.292, Z = −7.405, *p* < 0.001; high-quality food vs. low-quality food, Est = −1.130, *SE* = 0.302, Z = −3.745, *p* < 0.001; Figure 2a). As the variable presence/absence of the keeper was significant, we moved on with GLM_1b_ (target variable: anxiety-related behavior), to verify if keeper sex and their familiarity with bonobos could influence the target behavior. We found that the full model did not differ from the null model (likelihood ratio test: χ^2^ = 1.122; *df* = 2; *p* = 0.571).

**Figure 3:**
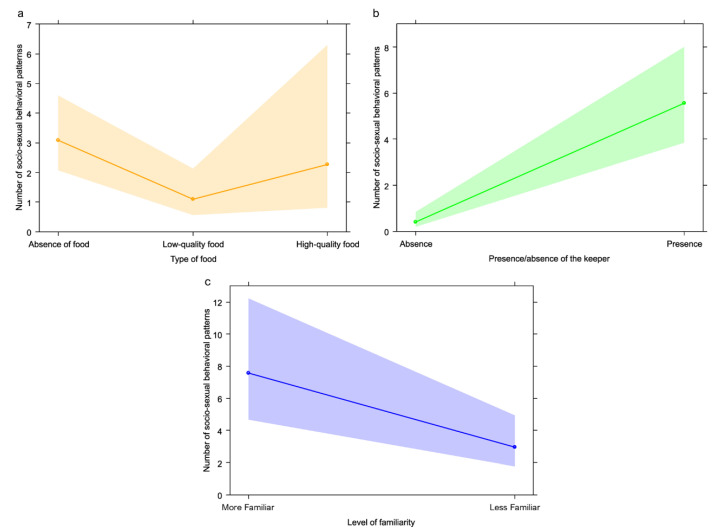
Effect plot of the variables with a significant effect on sociosexual behavior. The number of sociosexual behavioral patterns (y-axis) was (**a**) highest in absence of food (x-axis), (**b**) highest in presence of the keeper and especially (**c**) with more familiar keepers.

As concerns GLM_2a_ (target variable: sociosexual behavior), the full model significantly differed from the null model (likelihood ratio test: χ^2^ = 31.102; *df* = 3; *p* < 0.001), with a significant effect of all fixed factors (presence/absence of the keeper: *p* < 0.001; type of food: *p* = 0.007). We found the highest rates of sociosexual behavior in presence of keepers (Table 1; Figure 3b) and in absence of food (Table 1; Figure 3a; Tukey test: low-quality food vs. absence of food, Est = −1.038, *SE* = 0.387, Z = −2.679, *p* = 0.020; high-quality food vs. absence of food, Est = −0.309, *SE* = 0.551, Z = −0.561, *p* = 0.838; high-quality food vs. low-quality food, Est = 0.729, *SE* = 0.579, Z = 1.26, *p* = 0.412). We proceeded with GLM_2b_ (target variable: sociosexual behavior) on keeper features. We found a significant difference between the full and the null model (likelihood ratio test: χ^2^ = 6.586; *df* = 2; *p* = 0.037) with bonobos showing significantly higher rates of sociosexual behavior in presence (than in absence) of more familiar keepers (*p* = 0.007; Table 1; Figure 3c). Finally, we performed the third GLM (GLM2_c_; target variable: sociosexual behavior) including keeper identity as fixed factor. We found that the full model did not differ from the null model (likelihood ratio test: χ^2^ = 7.430; *df* = 3; *p* = 0.060).

**Figure 4:**
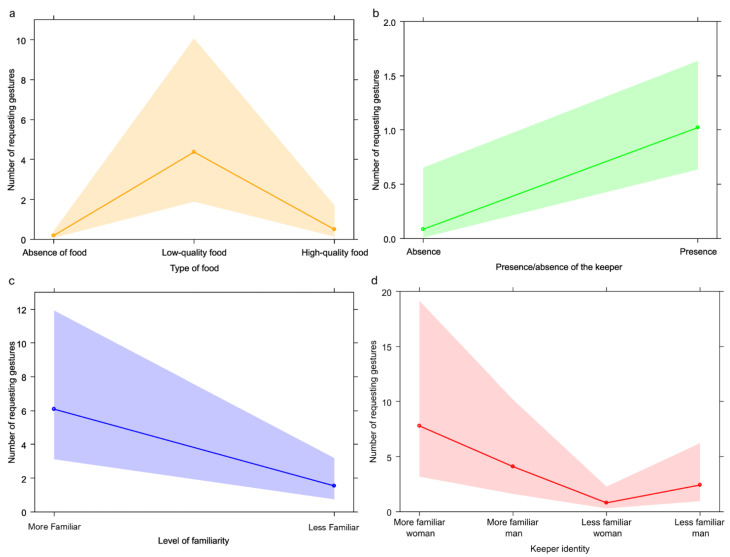
Effect plot of the variables with a significant effect on requesting gestures. The number of requesting gestures (y-axis) was (**a**) highest in presence of low-quality food (x-axis), (**b**) highest in presence of the keeper, (**c**) with more familiar keepers, and specifically (**d**) with the more familiar woman and man.

The full model with requesting gestures as target variable (GLM_3a_) significantly varied from the null model (likelihood ratio test: χ^2^ = 67.991; *df* = 3; *p* < 0.001), with a significant effect of all fixed factors (presence/absence of the keeper: *p* = 0.020; type of food: *p* < 0.001; Table 1). We found that the number of requesting gestures increased in presence of keepers (Figure 4b) and in presence of fruit and vegetables (Figure 4a; Tukey test: low caloric intake vs. absence of food: Est = 3.150, *SE* = 0.404, Z = 7.796, *p* < 0.001; high caloric intake vs. absence of food: Est = 0.981, *SE* = 0.608, Z = 1.614, *p* = 0.234 ; high caloric intake vs. low caloric intake: Est = −2.169, *SE* = 0.549, Z = −3.950, *p* < 0.001). We then proceeded with GLM_3b_ (fixed factors: keepers’ sex and keepers’ familiarity with bonobos) and we found that the full model significantly varied from the null model (likelihood ratio test: χ^2^ = 6.320; *df* = 2; *p* = 0.042). The GLM_3b_ revealed that in presence of the more familiar keepers, bonobos showed the highest number of requesting gestures (*p* = 0.005; Table 1; Figure 4c). Finally, we performed the GLM_3c_ (target variable requesting gestures) on keeper identity. We found that the full model significantly differed from the null model (likelihood ratio test: χ^2^ = 9.577; *df* = 3; *p* = 0.023). with a significant effect of the variable keeper identity (*p* = 0.001; Table 1; Figure 4d). We found that bonobos showed the highest number of requesting gestures to keeper 1 and 2 (the more familiar woman and the more familiar man; Tukey test: 2 vs. 1: Est = −0.648, *SE* = 0.641, Z = −1.012, *p* = 0.742; 3 vs. 1: Est = −2.294, *SE* = 0.696, Z = −3.297, *p* = 0.005; 4 vs. 1: Est = −1.165, *SE* = 0.650, Z = −1.792, *p* = 0.276; 3 vs. 2: Est = −1.645, *SE* = 0.702, Z = −2.345, *p* = 0.088; 4 vs. 2: Est = −0.517, *SE* = 0.656, Z = −0.787, *p* = 0.860; 4 vs. 3: Est = 1.129, *SE* = 0.710, Z = 1.589, *p* = 0.384).

**Figure 5:**
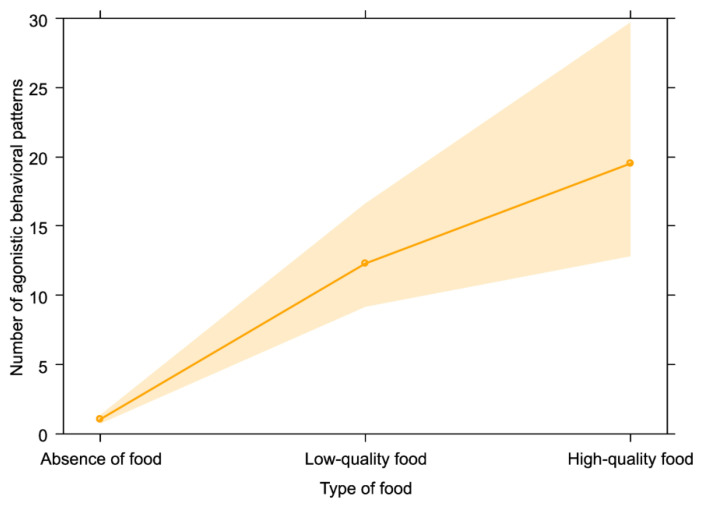
Effect plot of the variable with a significant effect on agonistic behavior. The number of agonistic behavioral patterns (y-axis) was highest in presence of high-quality food (x-axis).

The full model including play behavior as target variable (GLM_4_) did not differ from the null model (likelihood ratio test: χ^2^ = 5.452; *df* = 3; *p* = 0.142). As concerns the last model (GLM_5_, target variable: agonistic behavior), the full model was significantly different from the null model (likelihood ratio test: χ^2^ = 98.657; *df* = 3; *p* < 0.001). We found that bonobos showed the highest number of agonistic encounters in presence of food (*p* < 0.001; Table 1), especially when high-quality food was provisioned (Table 1; Figure 5; Tukey test: low-quality food vs. absence of food: Est = 2.495, *SE* = 0.228, Z = 10.952, *p* < 0.001; high-quality food vs. absence of food: Est = 2.956, *SE* = 0.271, Z = 10.904, *p* < 0.001; high-quality food vs. low-quality food: Est = 0.461, *SE* = 0.217, Z = 2.123, *p* = 0.084).

## 4. Discussion

Our results show that in the bonobo group housed at La Vallée des Singes the frequencies of the five behavioral categories considered in this study can be significantly influenced by food presence and quality as well as by keeper presence and familiarity. We found that: anxiety behaviors were more frequent in presence of the keeper (Prediction 1a not confirmed; Figure 2b) with no effect of familiarity (Prediction 1b confirmed), and in absence of food or in presence of low-quality food (Prediction 1c not confirmed; Figure 2a); sociosexual interactions were more frequent in presence of the keeper (Prediction 2a not confirmed; Figure 3b), especially if more familiar (Predictions 2b not confirmed; Figure 3c) and in absence of food (Prediction 2c not confirmed; Figure 3a); requesting gestures were more frequent in presence of the keeper (Prediction 3a confirmed; Figure 4b) especially when more familiar (Prediction 3b confirmed; Figure 4c) and in presence of low-quality food (Prediction 3c not confirmed; Figure 4a); play behavior was not affected by keeper presence/familiarity (Predictions 4a and 4b not confirmed) and by whether food was present or not (Prediction 4c confirmed); agonistic behavior was not influenced by keeper presence (Prediction 5a confirmed) and familiarity (Prediction 5b confirmed), but occurred most frequently in presence of high-quality food (Prediction 5c confirmed; Figure 5). 

### 4.1. Anxiety Behavior 

Contrary to our prediction, we found an increase in anxiety-related behaviors in presence of the keeper (Figure 2b), possibly because in our study the keeper was always associated with food provisioning. Our results show an increase of anxiety-related behaviors in absence of food (Figure 2a). The same pattern was found in stump-tailed macaques (*Macaca arctoides* [54]) where anxiety-related behaviors peak during the period preceding food distribution. A similar situation was found in François langurs (*Trachypithecus francoisi* [55]), which showed a peak of stress-induced vocalizations during the period that preceded food distribution (named pre-feeding). These results are probably related to the fact that captive animals are usually fed every day at the same time, which can generate food expectation anxiety [56]. 

### 4.2. Sociosexual Behavior 

Contrary to our predictions, sociosexual contacts were more frequent in presence of the keeper, especially when more familiar (Figure 3b, c). As occurred for anxiety behavior, the presence of the keeper was always associated with forthcoming food provisioning, which led to increased anxiety levels and the necessity to enact behaviors (such as sociosexual contacts [40]) that can help restore homeostasis. In contrast to anxiety behaviors, keeper familiarity appeared to be linked with higher levels of sociosexual interactions. Probably, more familiar keepers induced stronger food expectation given their habitual association with food and to the necessity of keeping anxiety under control by engaging in more sociosexual contacts. This explanation is supported by the fact that anxiety behaviors did not increase in case of more familiar keepers, as reported above (for Prediction 1b). An alternative interpretation could be that bonobos become more emotionally aroused when a familiar keeper is in front of them just because they share with this person a stronger social bond.

Moreover, sociosexual contacts were more frequent when the keeper was present and the food was unavailable, probably to reduce food expectation anxiety. Previous studies showed that in bonobos sociosexual contacts could increase when food was available, even though some sociosexual interactions were found to increase while waiting for food [33,41]. In our case, data were collected just before and during the feeding session (including pauses, in which food was not available) within a brief time slot, which probably led to the necessity of using sociosexual contacts as an arousal buffering mechanism. Interestingly, we found no significant difference in the frequency of sociosexual interactions between food absence and high-quality food presence. High-quality food (i.e., kibbles) generated more conflicts in the group (see Section 4.3) and a high level of sociosexual contacts can help buffer increased arousal levels, as suggested by the fact that anxiety behaviors were lower with high-quality food. Sociosexual contacts can be useful to reinforce social relationships and reduce aggression probability [35,57]. 

### 4.3. Requesting Gestures 

As expected, we found that the number of requesting gestures increased in presence of the keepers, especially when they were more familiar (Figure 4b–d). Indeed, imperative gestures are directed to another individual to catch their attention and request resources that are out of reach [24,25]. The increased frequency of requesting gestures in presence of a more familiar keeper is in line with a previous study carried out on bonobos by Gently and colleagues (2015) [38], who observed a higher number of requesting gestures toward familiar people. According to Gently et al. (2015) [38], the influence of familiarity on requesting gestures can be the result of a ‘conditional discrimination’ (a sort of operant conditioning) where the reinforcement of a neutral behavior (in this case the food request) depends not only on the positive outcome (food), but also on another stimuli (the keeper familiarity). Contrary to our expectation, low-quality food (i.e., fruit and vegetables) was associated with an increase in requesting gestures by bonobos (Figure 4a), compared to high-quality food (i.e., kibbles). This result is probably related to the different sizes of these food items: fruits and vegetables are provided in large pieces, whereas kibbles are small (peanut size). Therefore, pieces of fruits and vegetables are thrown in small numbers by the keeper and can be grabbed easily by bonobos. On the other hand, kibbles are thrown in larger numbers in the feeding area with no possibility for each single individual to withdraw them all. Hence, gestures to request kibbles were neither necessary nor useful to obtain more high-quality food. 

### 4.4. Play Behavior

Contrary to our prediction, neither the keeper nor the provisioned food was associated with a significant variation of the play behavior. As mentioned above, in our study the keeper was always associated with food and a function of play in bonobos is to face mild anxiety situations [33], such as periods around food. Previous studies reported an increase of play over long periods (up to 30 min) preceding food distribution in bonobos [33] and in case of high-quality food availability in different primate species (e.g., langurs [19] and squirrel monkeys [20]). In our case, the time window without food (either just before or during the feeding session) was very brief and with food ready to be distributed (or re-distributed, when bonobos had finished the first ration of food and were waiting for the next one). This may explain why no play difference was found in our study.

### 4.5. Agonistic Behavior 

As expected, the keeper (presence and familiarity) had no effect on aggressive levels in the bonobo group under study, possibly due to the high tolerance and low conflict rates of the species [30]. Moreover, high-quality food was associated with highest aggression levels (Figure 5), as it occurs in other primate species [31], possibly because of the increased value of the resource at stake. In the specific case of our study, the increased aggression rates may also be related to the increased time animals had to spend searching for kibbles by staying in close contact with other group members, with a consequent increase of the aggression risk. 

## 5. Conclusions

In conclusion, this study adds to the scientific knowledge about the impact that keeper (presence and familiarity) and food can have on the expression of behaviors possibly bearing a positive or negative emotional valence in captive bonobos. Our results suggest that bonobo welfare can be improved by: (i) favoring feeding sessions that follow a random time-schedule; (ii) preferring short latencies between keeper arrival and food distribution, in case of pre-scheduled feeding times (often planned for visitors) to reduce arousal/anxiety related behaviors; (iii) reducing pauses during feeding periods to reduce arousal/anxiety related behaviors; (iv) preferring always available, good quality (although not extremely highly caloric) food to reduce aggression risk (as also foreseen by the *rationed alternative diets*: e.g., [58]); (v) considering the social bonds that bonobos can establish with keepers, to enhance the anxiety buffering strategies that can be enacted when both environment and caregivers are familiar. 

On a broader perspective, this study shows that food is not the only variable to be included in the management equation to maximize the welfare of great apes. Owing to their complex cognitive and emotional abilities and phylogenetic closeness to our own species [26], great apes are more similar to humans than to other animal species and can be sensitive to similar environmental and social factors (e.g., keeper individual features, history, social relationships, etc.). This study paves the way for further investigation on the factors that should be taken into account to determine the extent to which great ape species management needs to be different from the management of other captive species. 

## Figures and Tables

**Table 1:** Full results on the effect of the keeper’s presence, the presence and type of food, the keeper/bonobo familiarity and the keeper identity on bonobos’ behavior.

Predictors	Estimates	SEM	CI_95_	Effect Size	*χ* ^2^	*p*
**GLM_1_** (Anxiety behaviors)	Full vs. null model: χ^2^ = 45.835; *df* = 3; *p* < 0.001					
(Intercept) ^a^	3.942	0.122	3.711; 4.189	^a^	^a^	^a^
Presence of keeper	0.522	0.171	0.186; 0.858	56.030	3.051	**<0.001**
Food type (low-quality food) ^b^	−1.031	0.187	−1.031; −1.395	72.949	−5.525	**<0.001**
Food type (high-quality food) ^b^	−2.161	0.292	−2.715; −1.565	26.007	−7.405	**<0.001**
**GLM_2a_** (Sociosexual behavior)	Full vs. null model: χ^2^ = 31.102; *df* = 3; *p* < 0.001					
(Intercept) ^a^	−0.624	0.348	−1.321; 0.057	^a^	^a^	^a^
Presence of keeper	2.625	0.425	1.803; 3.477	5.559	6.180	**<0.001**
Food type (low-quality food) ^b^	−1.038	0.387	−1.794; −0.264	3.082	−2.679	**0.007**
Food type (high-quality food) ^b^	−0.309	0.551	−1.317; 0.890	2.263	−0.561	0.575
**GLM_2b_** (Sociosexual behavior)	Full vs. null model: χ^2^ = 6.586; *df* = 2; *p* = 0.037					
(Intercept) ^a^	2.011	0.296	1.436; 2.662	^a^	^a^	^a^
Female keeper	0.023	0.348	−0.671; 0.715	4.793	0.067	0.947
Less familiar keeper	−0.935	0.349	−1.629; −0.241	7.559	−2.681	**0.007**
**GLM_2c_** (Sociosexual behavior)	Full vs. null model: χ^2^ = 7.430; *df* = 3; *p* = 0.060					
**GLM_3a_** (Requesting gestures)	Full vs. null model: χ^2^ = 67.991; *df* = 3; *p* < 0.001					
(Intercept) ^a^	−3.332	1.012	−6.211; −1.796	^a^	^a^	^a^
Presence of keeper	2.485	1.071	0.772; 5.417	1.1022	2.319	**0.020**
Food type (low-quality food) ^b^	3.150	0.404	2.384; 3.977	4.368	7.796	**<0.001**
Food type (high-quality food) ^b^	0.981	0.608	−0.212; 2.197	0.499	1.614	0.107
**GLM_3b_** (Requesting gestures)	Full vs. null model: χ^2^ = 6.320; *df* = 2; *p* = 0.042					
(Intercept) ^a^	1.889	0.414	1.012; 2.944	^a^	^a^	^a^
Female keeper	−0.164	0.490	−1.220; 0.868	3.341	−0.335	0.738
Less familiar keeper	−1.365	0.491	−2.418; −0.327	6.091	−2.782	**0.005**
**GLM_3c_** (Requesting gestures)	Full vs. null model: χ^2^ = 9.577; *df* = 3; *p* = 0.023					
(Intercept) ^a^	2.052	0.448	1.274; 3.069	^a^	^a^	^a^
More familiar man ^b^	−0.648	0.641	−1.939; 0.642	4.071	−1.012	0.312
Less familiar woman ^b^	−2.294	0.696	−3.695; −0.911	0.786	−3.297	**0.001**
Less familiar man ^b^	−1.165	0.650	−2.473; 0.141	2.428	−1.792	0.073
**GLM_4_** (Play behavior)	Full vs. null model: χ^2^ = 5.452; *df* = 3; *p* = 0.142					
**GLM_5_** (Agonistic behavior)	Full vs. null model: χ^2^ = 98.657; *df* = 3; *p* < 0.001					
(Intercept) ^a^	−0.154	0.220	−0.610; 0.257	^a^	^a^	^a^
Presence of keeper	0.256	0.296	−0.321; 0.844	2.643	0.864	0.388
Food type (low-quality food) ^b^	2.496	0.228	2.061; 2.956	19.545	10.952	**<0.001**
Food type (high-quality food) ^b^	2.956	0.271	2.439; 3.503	12.330	10.904	**<0.001**

^a^ Not shown as not having a meaningful interpretation. ^b^ These predictors were dummy-coded, with the reference category as follow: Food type: Absence of food; Keeper: More familiar woman.

## Data Availability

The data are available upon justified request from the corresponding authors.

## Supplementary Materials

The following supporting information can be downloaded at: https://www.mdpi.com/article/10.3390/ani13030410/s1, Table S1: Full group composition; Table S2: Description of the behaviors considered for the present study; Table S3: Energy value per food provided during feeding. References [59,60,61,62] are cited in the supplementary materials.
