# Supplementary material for: More Than Just Kibbles: Keeper Familiarity and Food Can Affect Bonobo Behavior
Source: Animals (Basel). 2023 Jan 26;13(3):410. doi: 10.3390/ani13030410 (PMC9913499; doi:10.3390/ani13030410)
Supplement: Supplementary file 1 [file animals-13-00410-s001.zip › animals-2077485-supplementary.pdf]

animals

MDPI

Article

Supplementary Files: More than Just Kibbles: Keeper Familiarity and Food Can Affect Bonobo Behavior

Marta Caselli [1], Emilio Russo [1], Jean-Pascal Guéry [2], Elisa Demuru [3,4,*,†] and Ivan Norscia [1,*,†]

[1] Department of Life Science and System Biology, University of Torino, Via Accademia Albertina 13, Torino 10123, Italy
[2] La Vallée des Singes, Romagne 86700, France
[3] Laboratoire Dynamique Du Langage, CNRS-UMR 5596, Université de Lyon, 14 avenue Berthelot, Lyon 69363, France
[4] ENES Bioacoustics Research Lab, CRNL, CNRS-UMR 5292, InsermUMR_S1028, Université de Saint-Etienne, 21, rue du Dr. Paul Michelon, Saint-Etienne 42100, France
* Correspondence: elisa.demuru@cnrs.fr (E.D.); ivan.norscia@unito.it (I.N.); Tel.:+33-0472-726546 (E.D.); +39-011-670-4547 (I.N.)
† I.N. and E.D. share the senior authorship.

Table S1. Full description of the group composition during the study period.

Subject	Sex	Year of birth
Daniela	Female	1968
Ukela	Female	1985
Ulindi	Female	1993
Diwani	Male	1996
David	Male	2001
Khaya	Female	2001
Lingala	Female	2003
Lucy	Female	2003
Kelele	Male	2004
Yahimba	Female	2009
Loto	Male	2009
Moko	Male	2012
Khalessi	Female	2012
Yuli	Female	2014
Swahili	Female	2014
Lokoro	Male	2015
Kymia	Female	2017

Table S2. Description of the behaviors considered for the present study (following Enomoto, 1990; Pollick and De Waal, 2007; Norscia et al., 2021; Demuru et al., 2022).

Agonistic Behavior	Description
**Avoid**	When an individual avoids interacting with another one, or when it changes its moving direction or goes far away from the latter.
**Bare Teeth**	Facial expression of fear where all teeth are exposed; usually associated with screaming.

**Fleeing**	An escape effectuated in an aggressive context.
**Screaming**	A scream vocalization of fear.
**Urinate**	An individual urinates for fear in an aggressive context.
**Defecation**	An individual defecates for fear in an aggressive context.
**Aggressive Bite**	An individual bites another one.
**Aggressive Brusque Rush**	An individual jumps on another one.
**Aggressive Crouching**	A crouching position assumed by an individual who is receiving an aggression, it is displayed to protect itself from the aggressor's hits.
**Aggressive Push**	An individual push another one by hands.
**Aggressive Pull**	An individual pulls another one by hands.
**Aggressive Slap**	An individual slaps another one by hands.
**Aggressive Stamping**	An individual jumps on another one with feet together.
**Charging Display**	It is composed of a series of behaviors (piloerection, run, facial expression, branch dragging, harm swinging, etc.) which generally are displayed by males to threaten other individuals, or to assess their dominance. In bonobo is common even within females.
**Chase**	An individual chases another one.
**Kick**	An individual kicks another one.
**Food Force Claim**	Two individuals get close to food and one of them win in taking it.
**PLAY BEHAVIOR**	**DESCRIPTION**
**Acrobatic play**	One or more individuals swing hanging from a support and/or jumping from tree to tree.
**Drag**	An individual drags another by grabbing him by the limbs.
**Full play face**	Mouth opening in which the upper teeth are also uncovered, carried out when play becomes very intense.
**Play face**	Mouth opening with the lower teeth exposed but the upper teeth covered by the lips.
**Grab**	An individual grabs someone by holding them tightly.
**Grab genitals**	An individual walks holding the genitals of the playmate.
**Jump**	An individual soars off the ground, remaining suspended in the air for a moment, with both feet raised.
**Kick**	An individual gives a hit with his feet to another individual.

**Moon walk**	An individual walks backward, generally keeping his or her gaze fixed on the other individual moving forward.
**Object steal**	An individual steals a game object from another individual.
**Pirouetting**	An individual performs revolutions on himself.
**Play bite**	Bite for fun, with not too much intensity.
**Play brusque rush**	An individual performs a pounce on another individual; generally performed by a young child on an adult or between two peers.
**Play crouching**	An individual squats in front of another individual.
**Play invitation**	An individual approaches a comrade and after hitting him runs away.
**Play pull**	An individual pulls a partner toward him or her with his or her hands or feet.
**Play push**	An individual pushes another with hands or feet.
**Play recovering a thing**	An individual steals an object from a play mate and then runs away only to be chased in turn.
**Play retrieve**	An individual restrains another by preventing them from moving away.
**Play run**	An individual chases another (may involve more than two individuals).
**Play slap**	An individual pats another individual on any part of the body.
**Play stamping**	An individual leaps over another individual with feet together.
**Play tourn around**	Two individuals chase each other; can also be done around another subject or object.
**Rough and tumble**	Fight and flight, generally done by infants and juveniles, but common among adults as well.
**Somersault**	An individual makes a leap by placing his hands or head on the ground, throwing his legs in the air and toppling over himself.
**Tickle**	An individual tickles another with his hands or feet or mouth.
**Tug of war**	Two animals compete for an object, and each pulls it toward itself.
**Wriggle**	An individual squirms free himself from the grasp of another individual.
**gallop**	An individual runs hopping toward another individual.
REQUESTING GESTURES	**DESCRIPTION**
**Reach out**	An individual stretches one hand toward another individual, extending the arm, wrist, hand, and fingers in a more or less horizontal

	position with the palm facing downward, sideways, and upward. There is no contact.
**SOCIO-SEXUAL CONTACTS**	**DESCRIPTION**
**Genito-Genital Rubbing (GGR)**	Two females in ventro-ventral, dorso-dorsal or ventro-dorsal position. Females rub their genital each other with lateral movements.
**Dorso-ventral copulation**	Mating complete with mounting, penetration and thrusting (pelvic thrusts) in which the female assumes a crouching position and receives the male from behind. It ends with ousting.
**Dorso-ventral mounting**	The male stands behind the female without penetrating her. Mounting can also be done by a male on another male.
**Inspecting**	An individual approaches another and inspects its genital area, touching and sniffing it. The action is performed by both males and females.
**Ventro-ventral copulation**	Mating complete with mounting, penetration, and thrusting (pelvic thrusts) in which female and male are in ventro-ventral contact.
**Ventro-ventral mounting**	The male stands over the female with ventro-ventral contact of the genitals, but without penetrating her.
**Rump-Rump rubbing**	Two individuals rub their genitals together by arranging their hindquarters in contact and rubbing them together.
**Sexual crouching**	Crouching position that the female may assume in mating from behind.
**Invitation**	This is a behavior that differs depending on whether it is performed by a male or a female. The male sits, stamps his feet on the ground, spreads his legs, swings sideways, back and forth, showing his erect penis. The female walks in front of the male, looks at him, stops, waits, and starts the sequence again. The female may lie down and assume the copulation position while looking at the animal (male or female) with which she intends to have sexual interaction.
**ANXIETY BEHAIVOR**	**DESCRIPTION**
**Yawning**	The yawning pattern involved mouth opening, with inhalation and a more rapid closing and exhalation.
**Self-scratching**	An individual rubs the skin with his hands or mouth.

Table S3. Energy value per food provided during feeding.

Low-quality food	kcal × 100g
Apples	~45
Apricots	~28
Carrots	~35
Broccolis	~27

Cauliflowers	~25
Onions	~25
Zucchini	~13
Cucumbers	~15
Endive	~10
Eggplant	~18
Peppers	~22
Tomatoes	~15
Celeriac	~22
High-quality food	kcal × 100g
Kibbles	~370

Disclaimer/Publisher's Note: The statements, opinions and data contained in all publications are solely those of the individual author(s) and contributor(s) and not of MDPI and/or the editor(s). MDPI and/or the editor(s) disclaim responsibility for any injury to people or property resulting from any ideas, methods, instructions or products referred to in the content.
